# Supplementary material for: What We Know about the Long-Term Risks of Hysterectomy for Benign Indication—A Systematic Review
Source: J Clin Med. 2021 Nov 16;10(22):5335. doi: 10.3390/jcm10225335 (PMC8622061; doi:10.3390/jcm10225335)

Supplementary figures and table

Table S1. Literature review search terms.

#	Searches	Results	Search strategy
Embase - ran on March 12, 2020			
1	(hysterectom* or oophorectom*).mp.	85658	Hysterectomy and UF terms
2	((leiomyoma or fibroma) and (uterine diseases or uterus)).mp.	9819	
3	((Uterine and (fibroma$ or fibroid$ or leiomyoma$ or myoma$ or fibromyoma$)) or (submucous fibroid$ or submucosal fibroid$ or Intramural fibroids)).mp.	22073	
4	1 and (2 or 3)	7935	
5	Prospective study/	585890	Observational/cohort/ clinical studies
6	(open label extension or OLE).mp.	5323	
7	clinical trial/	966894	
8	clinical study/	154759	
9	(electronic health record$ or EHR).mp.	29000	
10	(electronic medical record$ or EMR).mp.	70560	
11	patient record.mp. or Medical Records/	149646	
12	(real world or RWE).mp.	66551	
13	(dataset$ or data set$).mp.	252805	
14	prospective study/	585890	
15	(registry or register$).mp.	458842	
16	cohort analysis/	556749	
17	follow-up.mp.	1925435	
18	cohort.mp. or cohort analysis/	1007891	
19	case control.ti,ab.	159576	
20	(cohort adj (study or studies or analys*)).ti,ab.	298431	
21	((follow up or observational or uncontrolled or non randomi#ed or nonrandomi#ed or epidemiologic*) adj (study or studies)).ti,ab.	335438	
22	((longitudinal or retrospective or prospective or cross sectional) and (study or studies or review or analys* or cohort*)).ti,ab.	2098840	
23	or/5-22	5737389	
24	4 and 23	3090	Boolean combinations and exclusions
25	((long term adj8 (consequenc* or outcome or risk)) or associat* or sequala* or safety or adverse event* or mortality or morbidity).mp.	8256429	
26	24 and 25	1386	
27	limit 26 to (books or chapter or editorial or letter or note or "review" or short survey or tombstone)	131	
28	26 not 27	1255	
29	limit 28 to yr="2010 -Current"	896	
Medline - ran on March 12, 2020			
1	(hysterectom* or oophorectom*).mp.	52791	Hysterectomy and UF terms
2	((leiomyoma or fibroma) and (uterine diseases or uterus)).mp.	5187	
3	((Uterine and (fibroma$ or fibroid$ or leiomyoma$ or myoma$ or fibromyoma$)) or (submucous fibroid$ or submucosal fibroid$ or Intramural fibroids)).mp.	18445	
4	1 and (2 or 3)	4671	
5	Prospective study/	531064	Observational/cohort/ clinical studies
6	(open label extension or OLE).mp.	2775	
7	clinical trial/	521542	

8	clinical study/	3464	
9	(electronic health record$ or EHR).mp.	28334	
10	(electronic medical record$ or EMR).mp.	19300	
11	patient record.mp. or Medical Records/	67878	
12	(real world or RWE).mp.	39070	
13	(dataset$ or data set$).mp.	198979	
14	prospective study/	531064	
15	(registry or register$).mp.	282059	
16	cohort analysis/	256620	
17	follow-up.mp.	1299080	
18	cohort.mp. or cohort analysis/	623184	
19	case control.ti,ab.	122549	
20	(cohort adj (study or studies or analys*)).ti,ab.	203780	
21	((follow up or observational or uncontrolled or non randomi#ed or nonrandomi#ed or epidemiologic*) adj (study or studies)).ti,ab.	239190	
22	((longitudinal or retrospective or prospective or cross sectional) and (study or studies or review or analys* or cohort*)).ti,ab.	1391407	
23	or/5-22	3800112	
24	4 and 23	1478	Boolean combinations and exclusions
25	((long term adj8 (consequenc* or outcome or risk)) or associat* or sequala* or safety or adverse event* or mortality or morbidity).mp.	5769752	
26	24 and 25	610	
27	limit 26 to (books or chapter or editorial or letter or note or "review" or short survey or tombstone)	51	
28	26 not 27	559	
29	limit 28 to yr="2010 -Current"	294	
Cochrane databases - ran on March 12, 2020			
1	(hysterectom* or oophorectom*).mp.	8555	Hysterectomy and UF terms
2	((leiomyoma or fibroma) and (uterine diseases or uterus)).mp.	406	
3	((Uterine and (fibroma$ or fibroid$ or leiomyoma$ or myoma$ or fibromyoma$)) or (submucous fibroid$ or submucosal fibroid$ or Intramural fibroids)).mp.	2045	
4	1 and (2 or 3)	529	
5	Prospective study/	3	Observational/cohort/ clinical studies
6	(open label extension or OLE).mp.	3732	
7	clinical trial/	122	
8	clinical study/	0	
9	(electronic health record$ or EHR).mp.	1891	
10	(electronic medical record$ or EMR).mp.	2925	
11	patient record.mp. or Medical Records/	1151	
12	(real world or RWE).mp.	6459	
13	(dataset$ or data set$).mp.	8873	
14	prospective study/	3	
15	(registry or register$).mp.	70697	
16	cohort analysis/	956	
17	follow-up.mp.	277342	
18	cohort.mp. or cohort analysis/	62804	
19	case control.ti,ab.	7046	
20	(cohort adj (study or studies or analys*)).ti,ab.	14430	

21	((follow up or observational or uncontrolled or non randomi#ed or nonrandomi#ed or epidemiologic*) adj (study or studies)).ti,ab.	25130	
22	((longitudinal or retrospective or prospective or cross sectional) and (study or studies or review or analys* or cohort*)).ti,ab.	195567	
23	or/5-22	509201	
24	4 and 23	288	Boolean combinations and exclusions
25	((long term adj8 (consequenc* or outcome or risk)) or associat* or sequala* or safety or adverse event* or mortality or morbidity).mp.	611798	
26	24 and 25	169	
27	limit 26 to (books or chapter or editorial or letter or note or "review" or short survey or tombstone)	46	
28	26 not 27	123	
29	limit 28 to yr="2010 -Current" [Limit not valid in DARE; records were retained]	70	

Figure S1. Diagram of systematic review process.

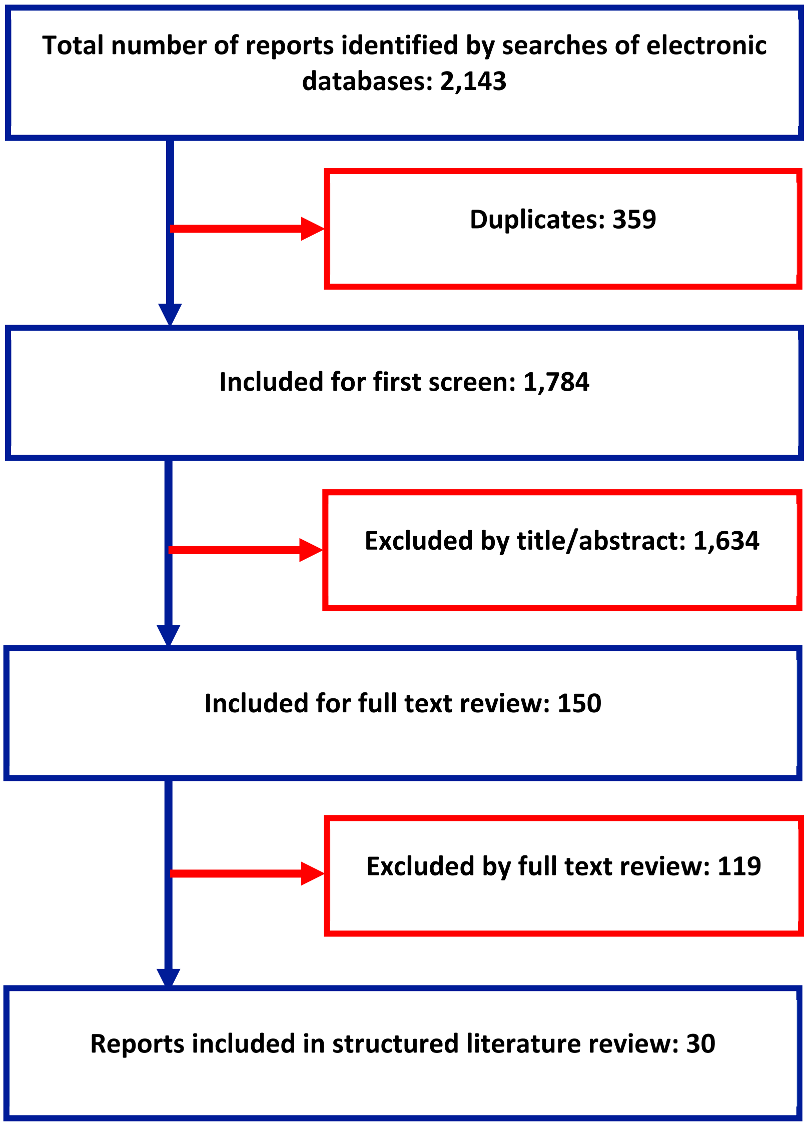

Supplement: Supplementary file 1 [file jcm-10-05335-s001.zip › jcm-1327540-supplementary.pdf]
